# Microscale ecology regulates particulate organic matter turnover in model marine microbial communities

**DOI:** 10.1038/s41467-018-05159-8

**Published:** 2018-07-16

**Authors:** Tim N. Enke, Gabriel E. Leventhal, Matthew Metzger, José T. Saavedra, Otto X. Cordero

**Affiliations:** 1 Department of Civil and Environmental Engineering, Massachusetts Institute of Technology, Cambridge, MA 02139 USA; 2 Department of Environmental Systems Science, ETH Zurich, Zürich, 8092 Switzerland

## Abstract

The degradation of particulate organic matter in the ocean is a central process in the global carbon cycle, the mode and tempo of which is determined by the bacterial communities that assemble on particle surfaces. Here, we find that the capacity of communities to degrade particles is highly dependent on community composition using a collection of marine bacteria cultured from different stages of succession on chitin microparticles. Different particle degrading taxa display characteristic particle half-lives that differ by ~170 h, comparable to the residence time of particles in the ocean’s mixed layer. Particle half-lives are in general longer in multispecies communities, where the growth of obligate cross-feeders hinders the ability of degraders to colonize and consume particles in a dose dependent manner. Our results suggest that the microscale community ecology of bacteria on particle surfaces can impact the rates of carbon turnover in the ocean.

## Abstract (web summary)

Particle-attached bacteria play a key ecosystem role by degrading complex organic materials in the ocean. Here, the authors use model marine microbial communities to show that community composition and interspecies interactions can significantly slowdown the rates of particle turnover in the environment.

## Introduction

Learning how the composition of ecological communities impacts their function is one of the central challenges in ecology^1–4^. In the case of microbes, this problem is particularly complex, not only because of the extreme diversity of taxa and genes that make up microbial communities, but also because community function depends on microscale processes that are hard to measure such as aggregation, dispersal, and cell–cell interactions^5^. A prime example of the link between microscale community ecology and large-scale ecosystem function is found in the biological turnover of particulate organic matter. In the marine environment, biopolymer particles formed by aggregation of fragments of decaying organisms, fecal pellets, and extracellular polysaccharides are degraded and consumed by heterotrophic bacteria that attach to particle surfaces and form dense microbial communities of large taxonomic and metabolic diversity^6–9^. Because particulate matter tends to sink in the water column, its degradation in the upper layers of the ocean where oxygen abounds is crucial to sustain the marine food web and prevent the sequestration of carbon and nitrogen into the deep sea^9–11^. Therefore, particle-attached microbial communities play a fundamental role by closing the loop of the global carbon cycle and maintaining the balance of nutrients in marine ecosystems. Although many physical aspects of the bacteria–particle interaction such as attachment or the effects of flow^12,13^ have been well characterized, the potential role that ecological interactions between microbes may play in controlling the dynamics of particle colonization and degradation—and thus the “mode and tempo” of the global carbon cycle—is much less clear.

Previous studies have suggested that ecological interactions between microbes can play a significant role in controlling the dynamics of community assembly on particles^14^. Competition for particle surface and thus primary resource access is likely to be strong among particle-attached bacteria and interference competition mediated by secondary metabolites can be a powerful strategy to deter competitors^15,16^. Moreover, over the time scales of particle turnover, trophic interactions mediated by byproducts of degradation and primary metabolism can strongly influence the overall dynamics of bacterial growth^17^. To release the carbon trapped in particulate matter, bacteria secrete hydrolytic enzymes—often regulated by quorum sensing signals^18^—that deconstruct complex biopolymers and release soluble sugars into the environment. The bioavailable sugars can in turn be taken up by nearby cells, thus unlocking a niche for cheaters that consume resources but do not contribute to degradation^17,19^. Likewise, byproducts of primary metabolism such as organic acids or amino acids that are released to the local environment can be consumed by cross-feeding bacteria that co-assemble on the particle. On chitin particles, these types of trophic interaction have been shown to lead to successional waves and invasion of secondary consumers, which eventually become the numerically dominant members of the community^17^. These findings led us to hypothesize that interactions across trophic levels at the microscale might alter the catabolism of chitin and consumption of byproducts, possibly affecting the rate of particle turnover and the conversion from particle to bacterial biomass.

To test this hypothesis, in this study we used an isolate collection obtained directly from particle-attached communities previously shown to colonize in microscale successions^17^. In brief, these communities were enriched on ~50 µm paramagnetic chitin hydrogel particles incubated in seawater from the coastal ocean (Nahant, MA, USA). Bacteria were isolated directly from the particles, resulting in a collection that includes taxonomic orders such as *Alteromonadales, Flavobacteriales, Rhodobacterales, Vibrionales,* and *Oceanospirillales*. Notably, the composition of our collection coincides well with the taxonomic profiles of natural chitinous marine particles collected at 200–500 m depth in the North Pacific gyre^20^. This overlap between our isolate collection and the taxonomic composition of natural particle-attached communities suggests that isolates obtained from model particles represent a relevant set of strains with which to study the effect of ecological interactions on particle turnover.

**Fig. 1:**
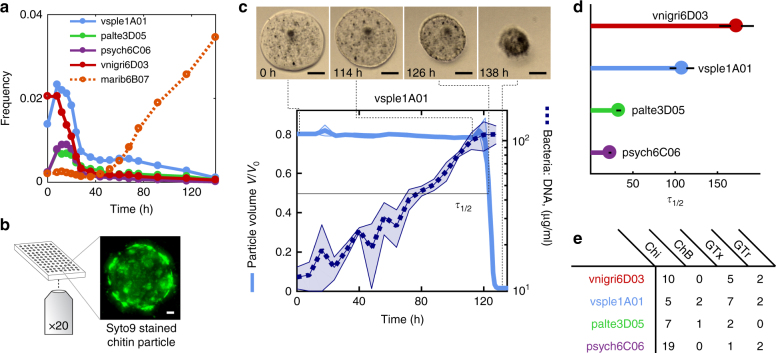
Particle degradation dynamics of bacteria isolated from chitin microparticles. **a** Culture-independent dynamics of four primary degraders (vsple1A01, palte3D05, psych6C06, and vnigri6D03) and a secondary consumer (marib6B07, dotted line). Trajectories shown depict dynamics of selected taxa in particle incubations with raw seawater. Data from ref.^ 16^. **b** In the laboratory, chitin particles immersed in bacterial suspensions are imaged at the bottom of microtiter plates for up to 240 h. The particle image corresponds to DNA stained palte3D05 after 24 h, showing the formation of bacteria microcolonies on the particle surface. Scale bar corresponds to 10 µm. For a 3D animation of the image, see Supplementary Movie 1. **c** Particle degradation kinetics (averaged over three different particles) and bacterial growth rate for vsple1A01 (averaged over three different incubations). The particle micrographs correspond to different time points during incubation with vsple1A01. Scale bar: 30 µm (see also Supplementary Movie 2). **d** Particle half-lives for the four different degraders tested with an inoculum of ~5 × 10^5^ cells per ml. **e** Number of gene copies of chitinases (Chi), chitin-binding proteins (ChB), GlcNAc-specific chemotaxis (GTx), and transport (GTr) genes. See also Supplementary Table 1

Bacterial isolates in our collection fall into two coarse-grained functional groups, defined on the basis of shared physiological characteristics and colonization dynamics^17^. The first group comprises primary degraders, which secrete chitinolytic enzymes, are motile, can grow rapidly on degradation byproducts, and belong to species that tend to appear early during particle colonization. The second group corresponds to secondary consumers, which in general do not secrete enzymes, cannot grow on chitin, grow poorly if at all on monomers, are not motile, and belong both to late and early successional species (Fig. 1a, Supplementary Fig. 1). Although secondary consumers cannot grow on chitin particles alone, they can reach 100–1000 fold higher abundance in the presence of primary degraders^17^ due to their ability to utilize carbon sources released by primary degraders during colonization.

Our goal in this study is to provide a quantitative description how particle degradation kinetics depend on the assembly of primary degraders and secondary consumers during particle colonization. To this end, we first studied how monocultures of primary degraders consumed particles by tracking changes in particle volume over time using high-throughput, high-resolution time-lapse microscopy (Fig. 1b, Supplementary Movie 1), and guiding our analysis with simple mathematical models of colonization and resource consumption. Subsequently, we assembled two-strain communities of primary degraders and secondary consumers and developed a quantitative phenomenological characterization of the impact of secondary consumers on degradation. Our results reveal that early colonizing taxa can differ significantly in their hydrolytic power to breakdown chitin, that particle degradation is limited by the number of enzyme-secreting bacteria that colonize the particle surface, and that secondary consumers effectively become parasites that increase in abundance at the cost of the primary degraders when co-colonizing on particle surfaces. Furthermore, the presence of parasitic secondary consumers can delay or even obstruct particle degradation. All these effects suggest that microscale community ecology on particle surfaces plays a major role in controlling community function by primarily slowing down resource turnover rates.

## Results

### Primary degraders display differences in hydrolytic power

We tracked the dynamics of particle consumption by measuring changes in particle volume over time, *V*(*t*), using high-throughput time-lapse microscopy of individual chitin microbeads. We chose an initial concentration of degrader cells of 5 × 10^5^ cells/ml—an upper-bound estimate of the concentration of degrading bacteria in coastal waters^21^—and quantified *V*(*t*) over a period of 240 h, for four primary degraders and four secondary consumers incubated in media with no carbon source other than the particle. As expected, secondary consumers, which are unable to use chitin as a carbon source, did not grow on particles in monoculture and therefore did not affect *V*(*t*) over the course of the 10-day time-lapse. For primary degraders, instead, *V*(*t*) was characterized by a long period of no detectable change, followed by an abrupt collapse (Fig. 1c, Supplementary Movie 2). Measurements of bacterial growth during degradation showed that bacteria grew steadily from the beginning of the incubation and that, despite no apparent change in particle volume, depolymerization was a continuous process that culminated in particle collapse (Fig. 1c, Supplementary Fig. 2). In some cases particles seemed to increase in volume immediately before collapse, possibly indicating that the degradation of cross-linked chitin in the hydrogel allowed water molecules to expand the matrix^22^. We interpreted the swelling-collapse transition as the point where a certain critical amount of polymer was degraded and used the time to reach this transition to quantify the ability of bacteria to consume particles. More precisely, we measured the hydrolytic power of degraders in terms of the particle half-life, *τ*_1/2_, i.e., the time it took for the particle to decrease to half its volume (see Methods).

We found a remarkable variation in *τ*_1/2_ among the four different primary degraders, despite the fact that all of these isolates appeared early on in the ecological succession on chitin particles (Fig. 1a). At an initial cell concentration of 5 × 10^5^ cells/ml for all primary degraders, particle half-lives varied from ~30 h for the fastest degrader (a strain of the genus *Psychromonas*, named psych6C06) to ~200 h for the slow degraders (a strain of *Vibrio nigripulchritudo* named vnigr6D03) (Fig. 1d). The large number of chitinase copies in psych6C06 (19 copies) suggested that gene dosage played a role in controlling the hydrolytic power of the strains. However, overall the differences between *τ*_1/2_ among primary degraders could not be clearly correlated to variation in gene content, suggesting instead that expression levels and the properties of extracellular enzymes played a more significant role. Gene content did, however, distinguish primary and secondary consumers: GlcNAc-specific phosphotransferase (PTS) transporters, chitin binding proteins, and GlcNAc-specific chemotaxis genes where all enriched among degraders compared to secondary consumers (Fig. 1e, Supplementary Table 1).

**Fig. 2:**
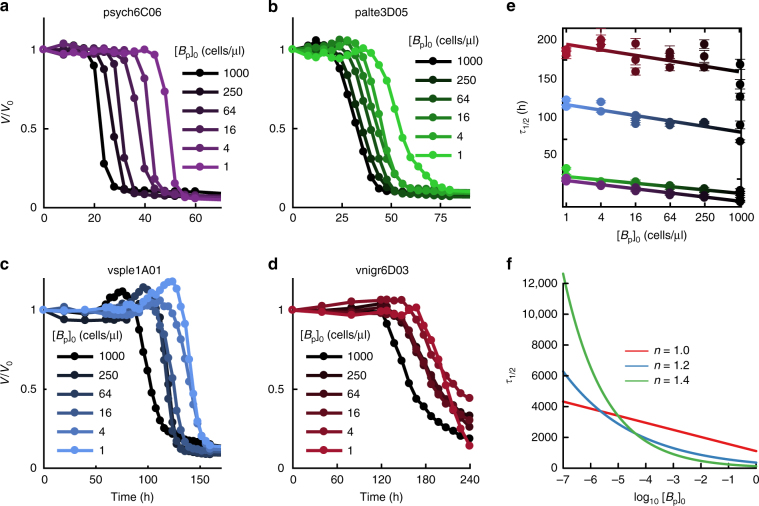
Effect of cell initial cell concentration on particle degradation kinetics. **a**–**d** Mean particle volume over time for primary degraders, over a range of initial inoculum concentrations, [*B*_p_]_0_. **e** Linear dependency between log[*B*_p_]_0_ and the particle half-life as predicted by Eq. (1) validates the simple model of degradation without cooperativity. **f** Prediction for log [*B*_p_]_0_ vs. half-life based on models with (*n* > 1) or without cooperativity (*n* = 1) for simulation parameters *a*_0_ = 0.01, *β* = 0.005, and *R*_0_ = 10^6^

Chitin degradation is intrinsically linked to the production of public goods such as chitinases and as such can be subject to cooperative growth dynamics^23^, i.e., a positive dependency between cell densities and growth or depolymerization rates. If cooperativity does play a role, half degradation times would be highly sensitive to cell numbers, increasing disproportionally in cases where cell load is low. To test the relevance of this phenomenon and, in general, to study how *τ*_1/2_ depended on initial conditions, we measured degradation kinetics as a function of the initial concentration of primary degrader, [*B*_p_]_0_, which until now was arbitrarily set to 5 × 10^5^ cells/ml. In addition, we guided our analysis with simple models of particle degradation and bacterial growth. To construct these models, we assumed that particle depolymerization was proportional to the density of bacteria, *B*. We studied two possibilities: that bacteria displayed cooperative growth kinetics, i.e., with growth rate proportional to *B*^*n*^ with *n* > 1 and that cooperativity played no significant role and growth and occurred at fixed, density independent per-capita rates. Assuming that *τ*_1/2_ depends linearly on the speed of depolymerization, under a scenario of no cooperativity we predict that *τ*_1/2_ should scale linearly with −ln[*B*_p_]_0_, whereas cooperative growth would make *τ*_1/2_ scale nonlinearly with ln[*B*_p_]_0_ (see Fig. 2f and Methods).

In agreement with the simplest model with no cooperativity, we find a linear relation between *τ*_1/2_ and ln[*B*_p_]_0_ (Fig. 2, Supplementary Table 2). This behavior implies that the particle half-life is controlled by simple mass action kinetics^24^ that—at least in the conditions of our experiment—are not influenced by cooperativity. More precisely, we find that *τ*_1/2_ is well described by the following expression:

1 $$\tau _{1/2}\sim t_0 - \frac{1}{\beta }{\mathrm{ln}}[B_{\mathrm p}]_0$$

where *t*_0_ is the intercept of the lines in Fig. 2e and represents a timescale to degradation that is intrinsic to each strain, *β* is the slope and represents the per-capita contribution to the degradation process and ln[*B*_p_]_0_ captures the effect of the primary degrader concentration in the local environment, akin to a chemical potential for the cell–particle reaction.

The relationship found in Eq. (1) shows that the turnover of particulate organic matter depends on the load of primary degraders in the milieu in a simple, predictable manner. The lack of an observed cooperativity suggests that the possible benefits that bacteria may derive from sharing hydrolysis products are effectively offset by local competition for resources between neighbors. Overall, our results indicate that variation in the composition and abundance of primary degraders can have a significant impact on the rate of particulate organic matter turnover.

### Secondary consumers behave as parasites

To understand how ecological interactions between primary degraders and secondary consumers influence particle degradation, we focused our analysis on two primary degraders and one secondary consumer. We chose the relatively “slow” degrader, vsple1A01 (Fig. 1c, d) a member of the *Vibrio splendidus* clade, the most abundant group of marine vibrios in coastal seawaters^25^, and the relatively “fast” degrader, *Pseudoalteromonas sp*. palte3D05, a common member of heterotrophic bacterioplankton communities^26,27^. Secondary consumers, or strains unable to degrade chitin, have previously been found to invade particle-attached communities and to become numerically dominant during community assembly^17^ (Fig. 1a). We focused our efforts on a secondary consumer cultivated from seawater-incubated chitin particles, a strain of the genus *Maribacter* (a type of marine *Flavobacteria*), that we here call marib6B07. As with other secondary consumers, marib6B07 is able to cross-feed when grown in coculture with degraders^17^. Interestingly, despite their inability to degrade chitin under laboratory conditions, the genomes of marib6B07 and the other secondary consumers all contain chitinases (e.g., marib6B07 has two). Yet, in contrast to degraders, none of these genomes contain genes for PTS transport of chitin monomers and only one out of the four consumers have genes for GlcNAc-specific chemotaxis and chitin-binding proteins (Supplementary Table 1). These differences in the genomes of primary degraders and secondary consumers suggest that their functional roles in the community may be determined by the interaction between multiple traits, such as the ability to swim toward breakdown products of chitin and to transport them into the periplasm.

**Fig. 3:**
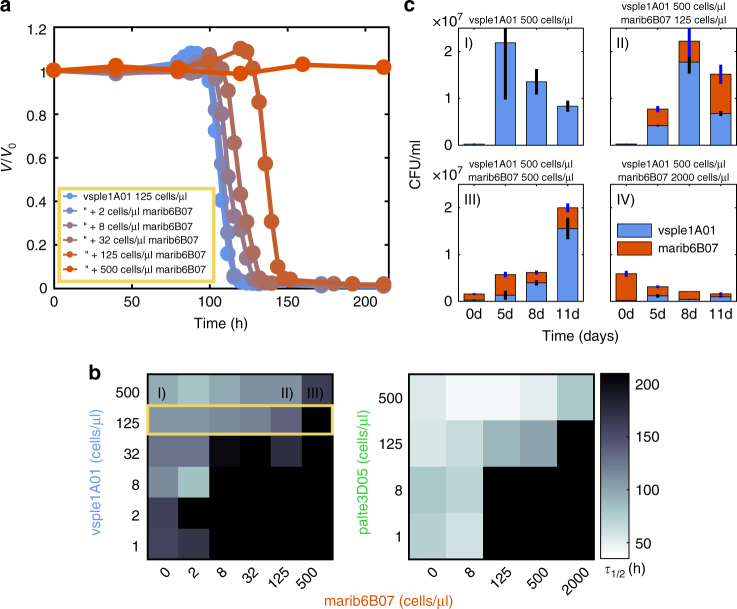
Secondary consumers inhibit degradation. **a** Particle degradation curves with different marib6b07 concentrations. At increasing concentrations of the secondary consumer the particle half-life increases disproportionally beyond the 220 h time limit. **b** Heat maps depict *τ*_1/2_ as a function of different primary degrader and secondary consumer inoculum concentrations and show that particle half-lives depend on the relative concentrations of primary degrader and secondary consumer cells. Color scale is the same for both heat maps. The highlighted row of the heat map corresponds to the degradation curves in **a**. For all degradation curves used for the heat maps see Supplementary Figs. 7–8. **c** CFUs of vsple1A01 and marib6B07 during coculture on chitin particles, showing that marib6B07 acts as a parasite reducing the yield of vsple1A01. (I) vsple1A01 in monoculture. Particle degradation observed at ~5 days. (II, III) Cocultures, particle degradation observed at ~8 and ~11 d, respectively. (IV) Coculture: no degradation observed (the error bars represent 1 standard deviation for *n* = 3 replicates). Decrease in CFU counts is due to loss of viability after degradation

Co-incubation of mari6B07 with vsple1A01 and palte3D05 showed that mari6B07 increased *τ*_1/2_ relative to primary degrader monocultures (Fig. 3a), implying that the cross-feeder impaired the ability of degrader populations to depolymerize the particle. To study this phenomenon in a quantitative manner, we measured how *τ*_1/2_ responded to changes in the initial concentration of secondary consumer, [*B*_s_]_0_, with the number of cells of the primary degrader fixed at a given concentration ([*B*_p_]_0_ ≈ 1.25 × 10^5^ cells/ml) (Fig. 3a). We found that for small values of [*B*_s_]_0_, *τ*_1/2_ increased roughly linearly, such that a onefold increase in the secondary consumer [*B*_s_]_0_ had approximately the same effect as a tenfold reduction of the primary degrader [*B*_p_]_0_ in monoculture.

Surprisingly, at a threshold [*B*_s_]_0_ we observed an abrupt increase in *τ*_1/2_, to the extent that particle degradation did not occur within the 240 h imaging period, suggesting that the population of primary producers might have been inhibited from colonization and/or growth. To investigate how this phenomenon depended on the composition of the two-strain community, we varied the abundance of the primary degraders, [*B*_p_]_0_ and secondary consumer [*B*_s_]_0_, in order to obtain degradation phase planes (Fig. 3b). The degradation phase planes show that complete inhibition did not depend on the total concentration of the secondary consumer, [*B*_s_]_0_, but on the ratio of secondary consumer to primary degrader *γ* = [*B*_s_]_0_/[*B*_p_]_0_, (Fig. 3b, c). For the slow degrader, vsple1A01, degradation was blocked at *γ* >~1, whereas for the fast degrader, palte3D05, degradation was blocked above a ratio of *γ* >~16, showing that the slow degrader was more sensitive to the inhibitory effects of secondary consumer marib6B07 than the fast degrader. This analysis indicates that the balance between the relative abundances of secondary consumers to primary degraders in the environment, in addition to the degradation kinetics of the primary consumer, may be an important parameter that dictates the turnover rates of carbon over short time scales (see Discussion).

Quantification of the abundance of each strain in coculture before and after particle degradation showed that the interaction between primary degrader and secondary consumer is parasitic, i.e., positive for the secondary consumer, negative for the degrader. CFU counts during the time course of degradation in cocultures of vsple1A01 and marib6B07 showed that primary degrader growth rate and yield were lower than in monoculture, and that the loss of degrader cells was compensated by the growth of secondary consumers (Fig. 3c). Secondary consumers doubled approximately five times by the time of particle collapse, in contrast to their zero doublings in monoculture. Notably, the total yield of the coculture was always equal or lower to the yield of the monoculture, highlighting the parasitic nature of the interaction. Similar results were observed with cocultures of palte3D05 and marib6B07 (Supplementary Fig. 3). Thus, secondary consumers, whose growth is facilitated by primary degraders, exert a negative feedback on degraders, limiting their ability to hydrolyze resources, and thereby potentially limiting their own growth.

**Fig. 4:**
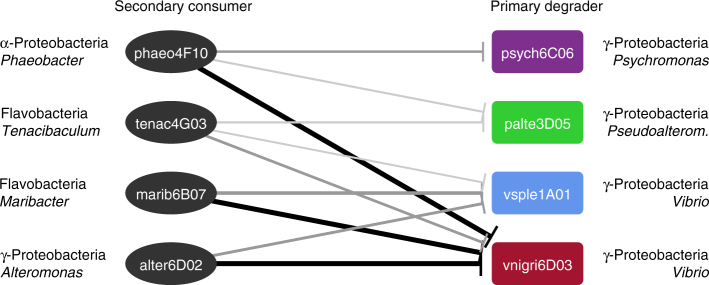
Degradation inhibition is specific to functional groups, not strains. The network depicts the effect of four different secondary consumers from diverse taxonomic origins on the characteristic particle half-life *τ*_1/2_ induced by different primary degraders. Network edge width is proportional to $\frac{{\tau _{1/2\, {\rm coculture}}}}{{\tau _{1/2\, {\rm monoculture}}}}$. Edges are drawn between secondary consumers and primary degraders when the mean (*n* = 3) half-life *τ*_1/2_ determined from the degradation curves of monoculture and cocultures was statistically different according to a one-way ANOVA. Black: complete inhibition, dark gray: *p* < 0.05, light gray: *p* < 0.1, respectively. See Supplementary Figs. 9, 10 and Supplementary Table 3 for raw data

Given the higher ratio of secondary consumer to degrader (*γ*) required to inhibit palte3D05 compared to vsple1A01, we hypothesized that “slow degraders” might be more susceptible to the detrimental effect of secondary consumers. To test this hypothesis as well as whether the observed parasitic interactions can be generalized to other primary degrader–secondary consumer pairs, we measured the effect of coculture at *γ* = 1 on particle degradation for all primary degraders (Fig. 1d) with four different secondary consumers (including marib6B07) of diverse taxonomic origins, all of which were co-isolated from the same chitin-attached communities. The results showed that while the fast degraders psych6C06 and palte3D05 were only mildly affected by coculturing with secondary consumers at *γ* = 1, the slow degraders vsple1A01 and vnigr6D03 were highly susceptible (Fig. 4). The slowest degrader, vnigr6D03, was in fact inhibited by all four secondary consumers, three of which caused total blockage of particle consumption. These data further indicate that parasitic interactions between degraders and consumers may not depend on specific taxa, but rather on the hydrolytic power of the degrader.

Consistent with the observation that interactions are not specific to strains or species but to functional roles (i.e., secondary consumer, primary degrader), we did not find evidence of chemical antagonism from secondary consumers to degraders. Agar plate assays designed to detect secreted inhibitory factors showed no interaction between the secondary consumer and primary degraders. Moreover, cocultures of vsple1A01 and palte3D05 with marib6B07 in liquid media supplemented with GlcNAc (the monomer of chitin) as sole carbon source showed no decrease in growth rates (Supplementary Fig. 4). This suggests that either an antagonistic factor is only secreted in the particle environment or, what is more likely, that the observed inhibition of primary degrader growth is based on interference with physical processes that only take place when resources are concentrated on particles (e.g., colonization and attachment).

## Discussion

Despite the significant efforts put into understanding the factors that drive the turnover of organic matter in the ocean^28,29^, the potential role that microbial interactions may play in this process has remained relatively unexplored. Our study leveraged a simplified model based on wild isolates that naturally colonize chitin particles to dissect this question. We provided evidence that both differences in primary degrader type and the ratio of primary degrader to secondary consumer can significantly alter particle degradation kinetics. Remarkably, we show that even in the ideal conditions of our experiments (no *N* limitation and high number of cells pregrown in rich media) particle turnover times can be as high as 200 h or more, that is, in the same range as the residence time of particles in the ocean’s mixed layer. Moreover, we showed interactions between primary degraders and secondary consumers lead to a significant increase in particle turnover times. We observed this phenomenon with different secondary consumers, suggesting that strain-independent processes such as blocking of the particle surface or alteration of resource gradients could mediate inhibition. The slowdown of the particle degradation kinetics imposed by the growth of secondary consumers is consistent with culture-independent data on colonization dynamics in natural seawater, which showed that secondary consumers invade particle-attached communities in a successional manner, displacing primary degraders and becoming numerically dominant in the community^17^. Taken together these results suggest that the microscale community ecology of particle-attached bacteria can play an important role in controlling rates of carbon turnover in the ocean. Further work will be needed to assess the precise mechanisms of interaction between primary degraders and secondary consumers on particle surfaces. Models with increased realism, including more complex particle compositions and predation by grazers or phages^14,30,31^ will be valuable to build a more complete picture of the community ecology processes that control the carbon cycle in the ocean.

## Methods

### Bacterial culturing conditions

Bacterial strains used in this study were previously isolated from model chitin particles^17^. Strains were streaked from glycerol stocks onto Marine Broth 2216 (Difco #279110) 1.5% agar (BD #214010) plates. After 48 h, single colonies were transferred to 2 ml liquid Marine Broth 2216 and incubated at room temperature, shaking at 200 rpm. Saturated liquid cultures were harvested after 48 h by centrifugation for 8 min at 3000 rpm (Eppendorf 5415D, Rotor F45-24-11) and washed two times with Tibbles–Rawling minimal media (see Supplemental material of Ref. 17 for a detailed recipe). Optical density (OD) 600 nm was determined in 200 µl (50 µl culture and 150 µl minimal media) in a clear 96-well plate (VWR 10062-900) with a spectrophotometer (Tecan Infinite F500). Cell numbers were normalized to the desired initial concentrations using a three-point linear calibration between OD 600 nm and direct cell counts determined with a Guava easyCyte Benchtop Flow Cytometer for each strain.

### Particle degradation experiments

Particle degradation experiments were performed in clear 96-well plates (VWR 10062-900). Each well contained 180 µl Tibbles–Rawling minimal media, bacterial cells at defined concentrations prepared as described above, and approximately 100 chitin magnetic beads (New England Biolabs #E8036L). Before being used in the experiments, the chitin magnetic beads storage buffer was removed using a neodymium magnet (McMaster-Carr #5862K38) to retain the beads. Beads were washed twice in Tibbles–Rawling minimal media and size selected using 100 µm and 40 µm strainers (VWR, #10199-658 and #10199-654, respectively).

For Fig. 1b, the colonized particle was stained in the well after 24 by adding Syto9 (Thermo Fisher, S34854), 500 nM final concentration for 1 h at room temperature in the dark. Microscopy was performed on an EVOS FL Auto Imaging System (Fisher #AMAFD1000) using a GFP lightcube (Thermo Fisher AMEP4651) and a 20× fluorite, long working distance objective (Fisher #AMEP4682, NA 0.40, WD 3.1 mm) and the software’s (revision 31201) *Z*-stack function. 3D-reconstruction was done using the ImageJ distribution Fiji (ImageJ 1.51N).

### Time-lapse imaging

Phase-contrast time-lapse images were acquired with an EVOS FL Auto Imaging System (Fisher #AMAFD1000) using the EVOS software (revision 31201) and a 20× fluorite, long working distance, phase-contrast objective (Fisher #AMEP4682, NA 0.40, WD 3.1 mm). Images were manually focused for each particle to capture the maximum cross-section area (see Fig. 1c, upper panel). Time lapses ran a maximum of 240 h, with images acquired every 2 h. To minimize evaporation effects, culturing plates were wrapped in para film during the time-lapse experiments and outer wells filled with 200 µl water.

### Image processing and volume quantification

Phase-contrast images were analyzed using the ImageJ distribution Fiji (ImageJ 1.51N). A polygonal shape was manually drawn around the particle to determine the area of the particles’ cross-section. To convert from cross-section area in square pixel (1 pixel = 0.4545 µm) to volume (in µm^3^), we assumed a spherical shape of the particles. Volumes were normalized to initial volume at *t* = 0 h to account for variation in particle sizes. In order to estimate the particle half-life, we fitted a sigmoidal function $\frac{1}{{1 + {\mathrm e}^{\left( {k\left( {x - \tau _{1/2}} \right)} \right)}}}$ using MATLAB (Version R2016b) and the “fit” function with initial values for *k* (0.5) and *τ*_1/2_ (initial estimates vary for each strain), constraining both variables to positive values (see also Supplementary Fig. 5 for examples of sigmoidal fits to the data).

### Coculture experiments

Cell counts were obtained by sampling 100 µl from 96-well culture plates (inoculated with 170 µl minimal media, 2 × 10 µl of the normalized bacterial culture, and 10 µl particles as described above). Imaging was performed as described above. For CFU counts, samples were vortexed thoroughly to detach cells from particles and 10 µl were plated in 10^−2^ and 10^−3^ dilutions in replicates on MB2216 agar plates using rattler beads (Zymo S1001). After 72 h, colonies were counted to obtain CFUs.

### DNA quantification

To quantify DNA as a proxy for biomass from monocultures in 96-well plates, wells were mixed thoroughly by pipetting and 100 µl of each well (including the particles) were sampled and frozen at −20 °C for subsequent analysis. Cells were lysed by thawing and boiling (95 °C, 10 min) 10 µl of each sample. Lysed samples were diluted 1:10 in TE buffer and quantified using Quant-it pico green (Fisher # P7589) standard protocols.

### Modeling of particle half-life for noncooperative degraders

The degradation of a chitin particle by bacteria can be modeled in a simple way by taking into account two processes: free-living bacteria attach to the particle surface at a rate, *a*, proportional to their planktonic concentration, [*B*]_0_, such that *a* = *a*_0_ [*B*]_0_, where *a*_0_ is the attachment rate per bacterial cell, and attached cells degrade the particle at a rate *p*, and chitin monomers are converted to bacterial biomass at a conversion factor *r*. Note, that the conversion factor *r* may take into account the loss of monomers to the environment. This results in a set of differential equations for the amount of bacterial biomass, *B*(*t*), and the total amount of particle, *R*(*t*),

2 $$\frac{{{\mathrm d}B}}{{{\mathrm d}t}} = a + rpB,\,B\left( 0 \right) = 0$$

3 $$\frac{{{\mathrm d}R}}{{{\mathrm d}t}} = - pB,\,R\left( 0 \right) = R_0$$

In the above parametrization, the degradation of a chitin particle is described by four independent parameters: the total size of the particle, *R*_0_; the attachment rate of bacteria, *a*; the biomass conversion rate, *r*; and (iv) the degradation rate; *p*. By rescaling the total number of bacteria by the growth rate, we obtain a more canonical parametrization: let *b*(*t*) = *B*(*t*)/*r*, *α* *=* *a*/*r*, and *β* = *rp*, then,

4 $$\frac{{{\mathrm d}b}}{{{\mathrm d}t}} = \alpha + \beta b,\,B\left( 0 \right) = 0$$

5 $$\frac{{{\mathrm d}R}}{{{\mathrm d}t}} = - \beta b,\,R\left( 0 \right) = R_0$$

which, for initial conditions *b*(0) = *B*(0) = 0 and *R*(0) = *R*_0_, are solved by the equations

6 $$b\left( t \right) = \frac{\alpha }{\beta }\left( {{\mathrm e}^{\beta t} - 1} \right)$$

7 $$R\left( t \right) = R_0 - \frac{\alpha }{\beta }\left( {{\mathrm e}^{\beta t} - 1} \right) + \alpha t$$

From Eq. (6) the time required to fully degrade the particle can be found by numerically solving the transcendental equation for *R*(*T*) = *R*_0_. Additional analytical insight can be gained, however, by assuming that attachment is slow compared to growth, and hence *R*(*T*) ≈ *R*_0_ − *b*(*T*). Furthermore, we additionally assume that *rpT* ⪢ 0 and, hence ln(e^*rpT*^−1) ≈ *rpT*. This leads to a simple expression for the total degradation time, *T*, required to fully degrade a particle,

8 $$T = \frac{1}{\beta }\left( {\ln R_0 + \ln \beta + \ln \frac{1}{\alpha }} \right)$$

Hence, the degradation time, *T*, depends linearly on the logarithm of the attachment rate *a*, and hence the planktonic concentration of bacteria (see Supplementary Fig. 6).

There is an intuitive interpretation of the degradation time from Eq. (7): the increase in bacteria on the particle (Eq. 3) can be subdivided into two regimes. In the first regime, *b* ≈ 0, such that $\frac{{{\mathrm d}b}}{{{\mathrm d}t}} \approx \alpha$ and attachment dominates. In this regime, the number of bacteria on the particle increases linearly with time, *b*(*t*) ≈ *αt*. In the second regime, once a certain density of bacteria has been reached, i.e., *b*(*t*) ⪢ *α*/*β*, growth takes over as the dominant contributor, such that $\frac{{{\mathrm d}b}}{{{\mathrm d}t}} \approx \beta b\left( t \right)$, and *b*(*t*) ~ e^*βt*^. These two regimes correspond to the terms $\ln \frac{1}{\alpha }$ and ln*β* in Eq. (7). Note, that in this formalism the number of bacteria could increase indefinitely. In reality, however, it is plausible that there exists a maximum carrying capacity for bacteria on the particle. Any effect of total carrying capacity will only kick in in the second regime, and will thus not affect the contribution of attachment to the degradation time.

### Expected half-life for cooperative degraders

To calculate half-lives in the presence of cooperative growth we used a simple extension to the model,

9 $$\frac{{{\mathrm d}b}}{{{\mathrm d}t}} = \alpha + \beta b^n,\,b\left( 0 \right) = 0$$

10 $$\frac{{{\mathrm d}R}}{{{\mathrm d}t}} = - \beta b,\,R\left( 0 \right) = R_0$$

We solve these equations numerically to find the time it takes to consume half of the resources, *R*(*τ*_1/2_) = *R*(0)/2, as a function of *α* (*R*(0) = 10^5^, *β* = 0.005). Simulations were performed in R using the deSolve package.

### Genome sequencing and annotation

Genomes were sequenced on an Illumina MiSeq instrument, assembled with the CLC Assembly Cell and annotated using the RAST^32^ annotation pipeline with default parameters.

### Data availability

Sequence data that supports the findings of this study have been deposited in the NCBI database under Bioproject # PRJNA414740 with genome accession numbers “PIZM00000000”, “PDUR00000000”, “PDUS00000000”, “PIZL00000000”, “PDUT00000000”, “PDUV00000000”, “PDUU00000000”, “PIZK00000000”. All other data supporting the findings of this study are available from the corresponding author upon request.

## Electronic supplementary material

Supplementary Information

Description of Additional Supplementary Files

Supplementary Movie 1

Supplementary Movie 2
