# Supplementary material for: Microscale ecology regulates particulate organic matter turnover in model marine microbial communities
Source: Nat Commun. 2018 Jul 16;9:2743. doi: 10.1038/s41467-018-05159-8 (PMC6048024; doi:10.1038/s41467-018-05159-8)
Supplement: Supplementary file 1 — Supplementary Information [file 41467_2018_5159_MOESM1_ESM.pdf]

Supplementary Information

Micro-scale ecology regulates particulate organic matter turnover in model marine microbial communities

Tim N. Enke[1,2], Gabriel E. Leventhal[1], Matthew Metzger[1], José T. Saavedra[1] and Otto X. Cordero[1]

[1] Department of Civil and Environmental Engineering, Massachusetts Institute of Technology

[2] Department of Environmental Systems Science, ETH Zurich

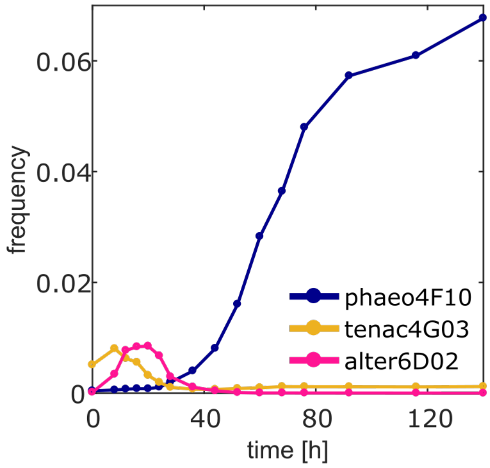

Supplementary Figure 1: Culture independent dynamics of secondary consumers phaeo4F10, tenac4G03 and alter6D02. Trajectories shown depict dynamics of selected taxa from particle incubations with raw seawater, where other taxa were present. Data from [16].

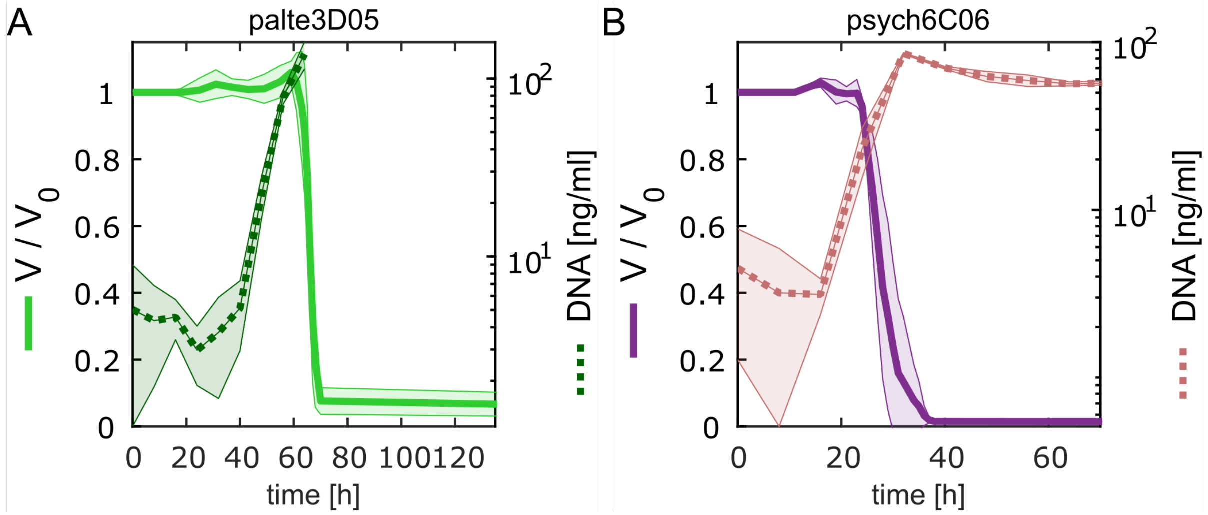

Supplementary Figure 2: **Degradation dynamics and bacterial growth for palte3D05 and psych6C06.** Particle volume over time normalized to initial volume (solid line) and bacterial abundance as measured by the amount of DNA extracted from ~100 particles at different points of colonization (dashed line). The standard deviation of measurements was calculated using three replicate particles from the same well, and three different bulk incubations for DNA.

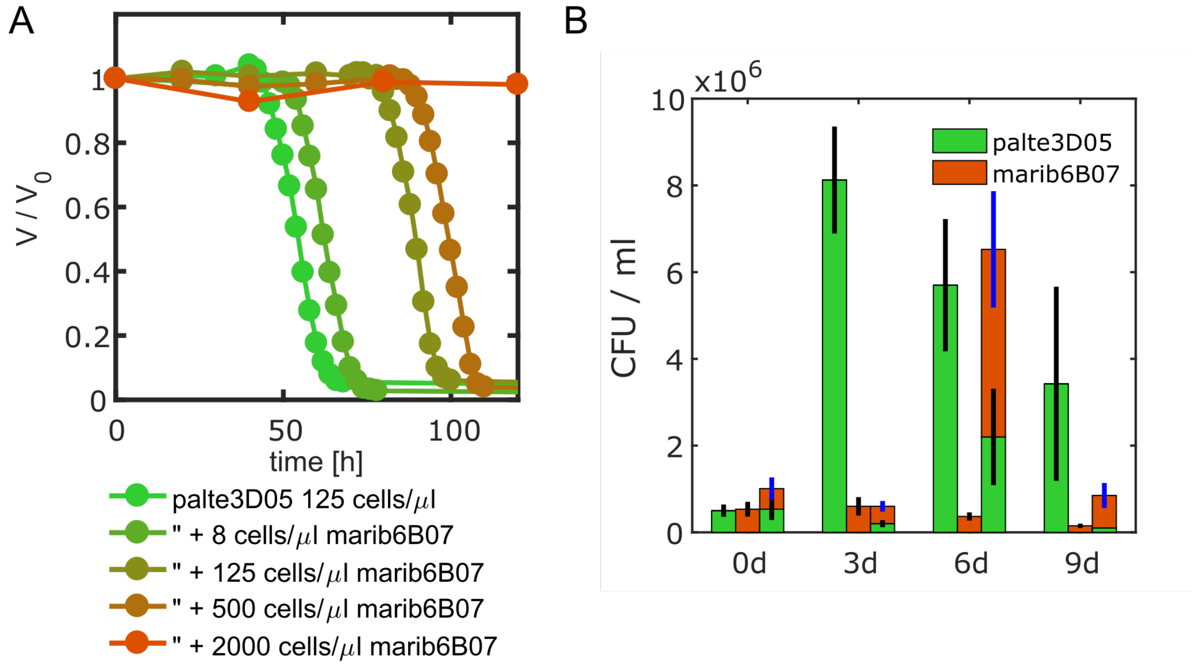

Supplementary Figure 3: Secondary consumer can inhibit degradation of primary degrader palte3D05. **A)** Particle degradation curves with different marib6b07 concentrations. At increasing concentrations of the secondary consumer the particle half-life increases disproportionally beyond the 220 h time limit (endpoint not shown). **B)** CFUs of palte3D05 (500 cells / µl) and marib6B07 (500 cells / µl) during mono- (first two bars for each time point) and co-culture (third, stacked bar) on chitin particles, showing that palte3D05 growth in mono-culture peaks at ~3d and marib6B07 cannot grow in mono-culture. Marib6B07 grows “at the expense” of palte3D05’s yield and delays peak growth and particle degradation which occurred at ~6d. Black error bars correspond to palte3D05, blue error bars to marib6B07, respectively (both depict standard deviation for n=3 replicates). Decrease in CFUs is due to loss of viability after degradation.

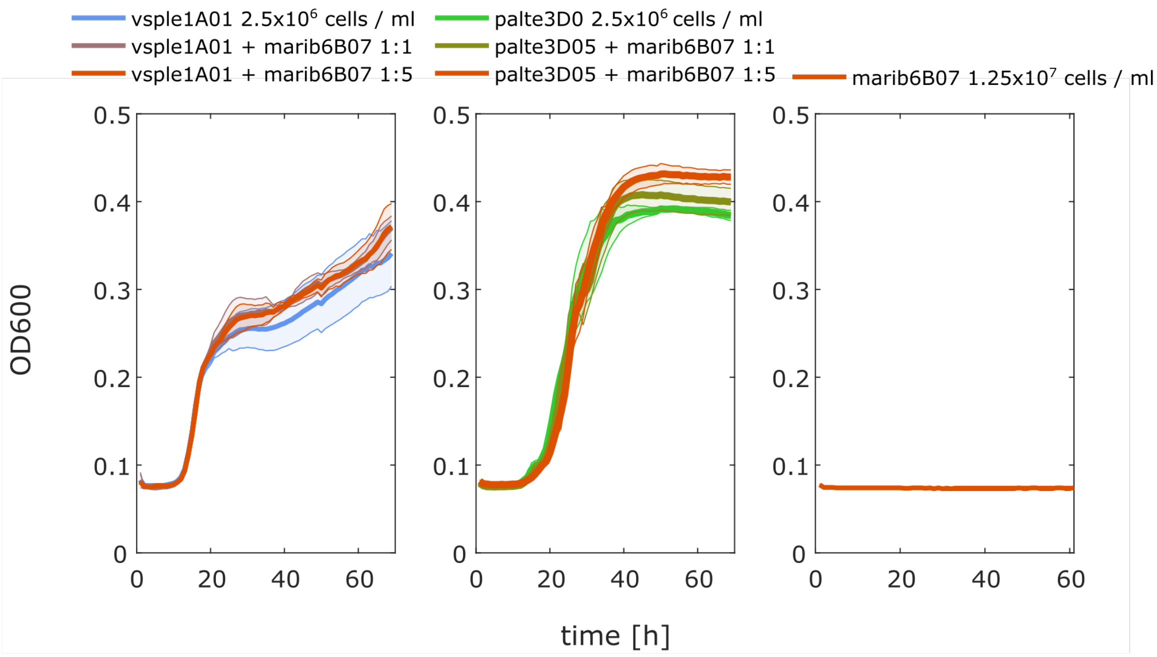

Supplementary Figure 4 Growth of secondary consumer marib6B07 in co-culture with primary degrader vsple1A01 (left panel), palte3D05 (middle), and in monoculture (right panel) on 0.1 % GlcNAc (*N*-Acetyglucosamin, chitin monomers). Co-cultures are in 1:1 and 1:5 ratios of primary degrader to secondary consumer, as indicated above the panels.

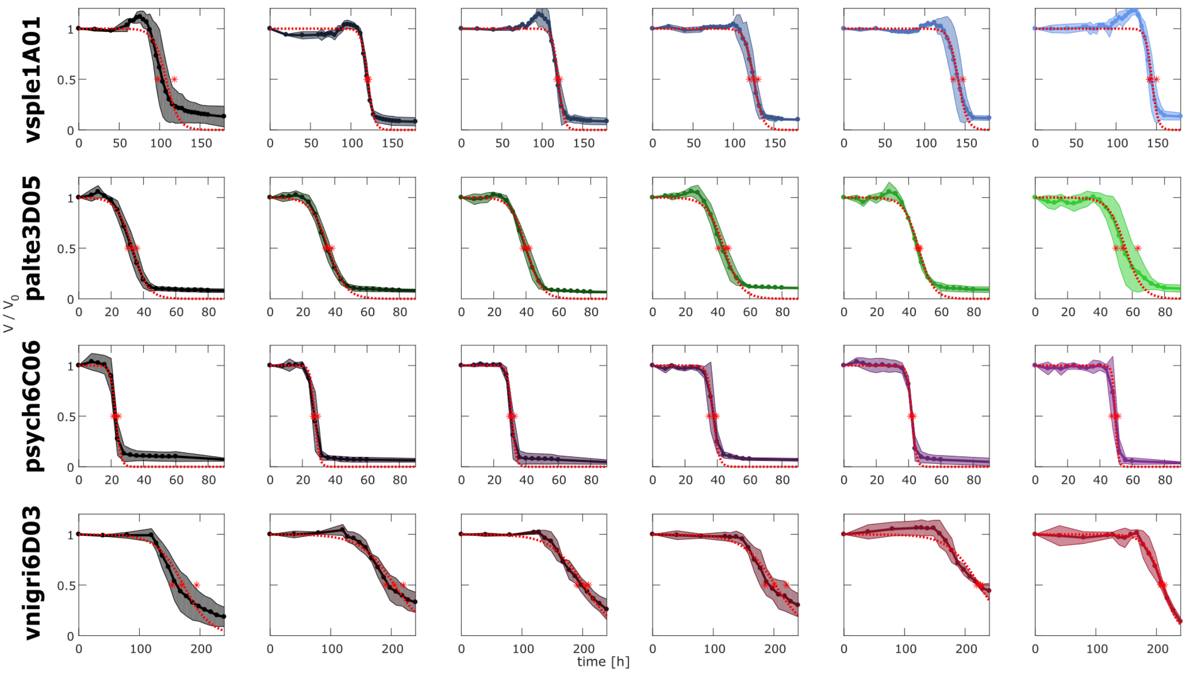

Supplementary Figure 5: Particle volume over time for different initial concentration of primary degraders. Data corresponds to Fig 2 A-D. Shown are quantified, normalized particle volumes for four primary degraders and six initial cell concentrations (from left to right: 2^{10}, 2^8, 2^6, 2^4, 2^2, 2^0 cells / µl). Solid line: mean, shaded area: standard deviation of n=3 replicate particles. Dashed red line: fit of a sigmoidal function to the mean; red asterisk: inferred tau for the three single replicates (see methods).

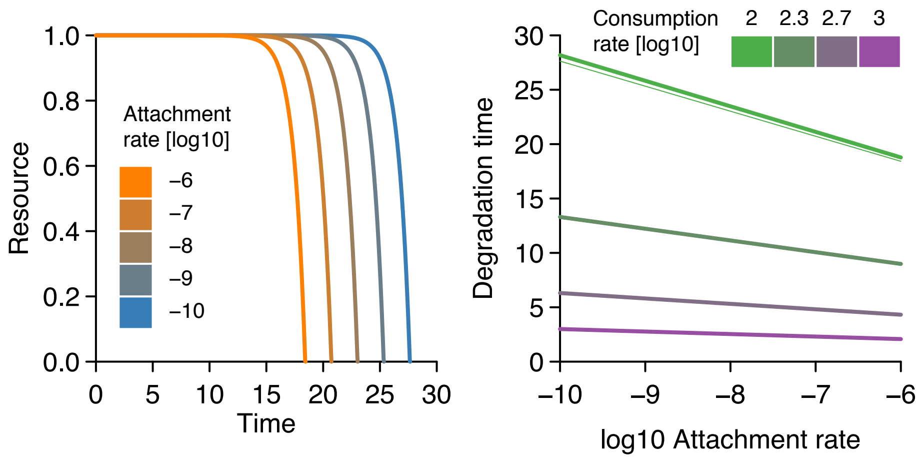

Supplementary Figure 6: Left panel: degradation dynamics predicted by equations 1-6. Attachment rate is the product of the per-cell attachment rate and the number of initial bacteria in the medium. Right panel: particle half-lives as a function of attachment rates for populations with different hydrolytic powers

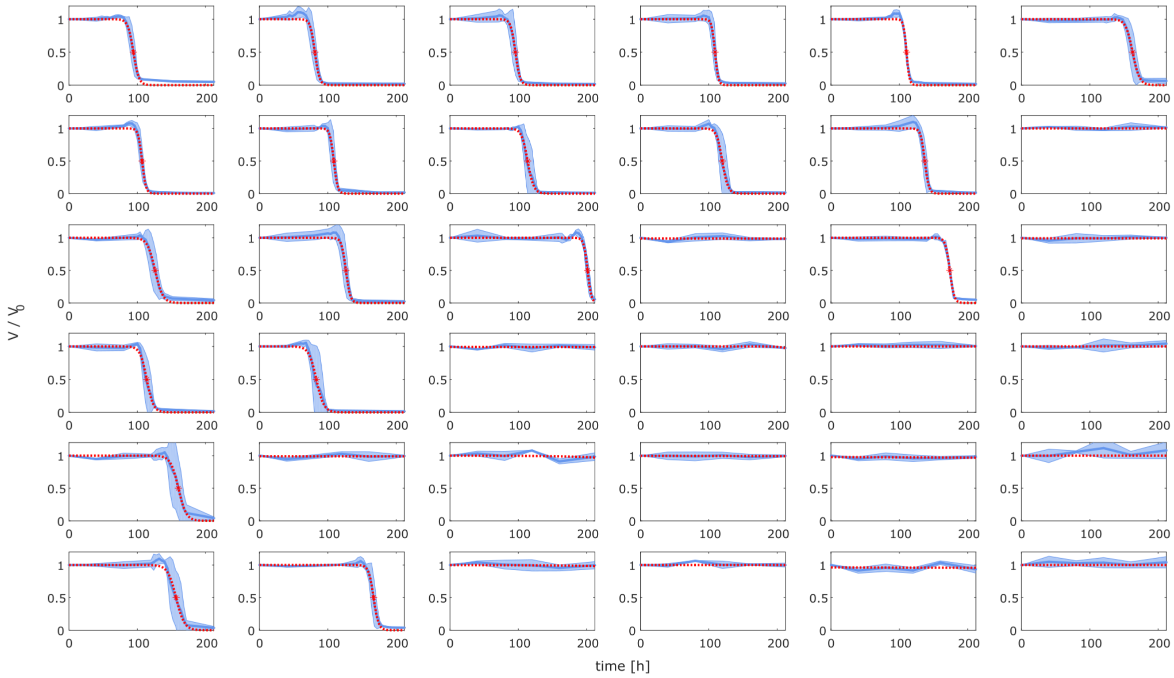

Supplementary Figure 7: Particle volume over time for different initial concentrations of primary degrader vsple1A01 and secondary consumer marib6B07. Data corresponds to Fig 3C, left heatmap. Shown are quantified, normalized particle volumes for all fields of the heat map in the same arrangement. Solid line: mean, shaded area: standard deviation of n=3 replicate particles. Dashed red line: fit of a sigmoidal function to the mean; red asterisk: inferred tau from mean as shown in heat map (see methods).

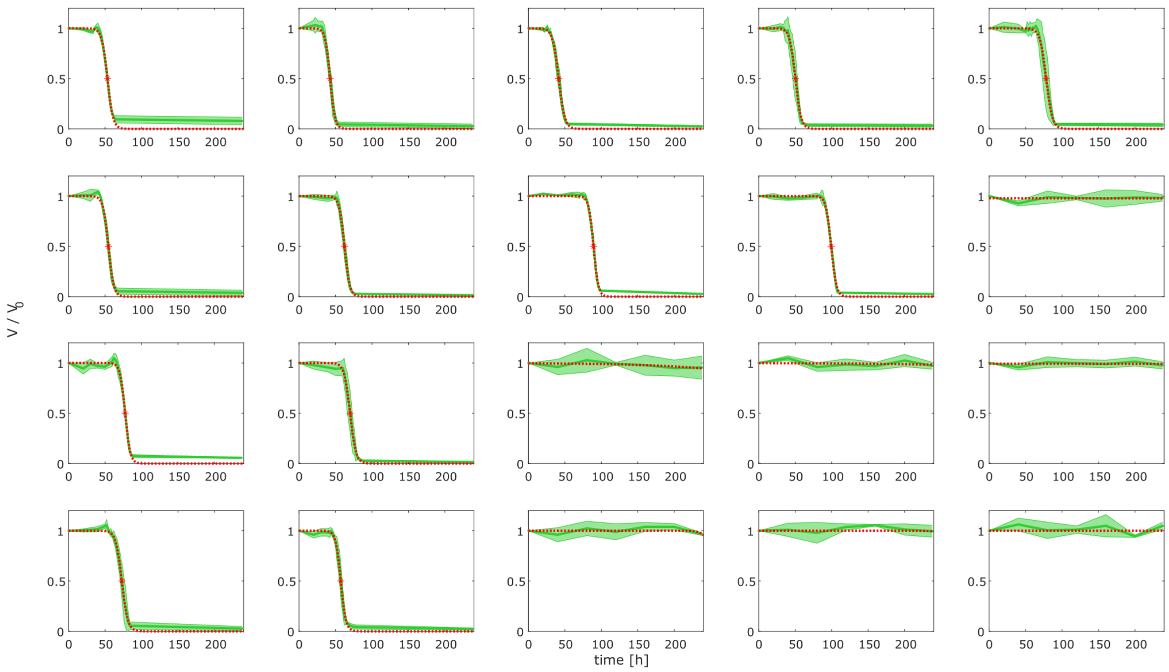

Supplementary Figure 8: Particle volume over time for different initial concentrations of primary degrader palte3D05 and secondary consumer marib6B07. Data corresponds to Fig 3C, right heatmap. Shown are quantified, normalized particle volumes for all fields of the heat map in the same arrangement. Solid line: mean, shaded area: standard deviation of n=3 replicate particles. Dashed red line: fit of a sigmoidal function to the mean; red asterisk: inferred tau from mean as shown in heat map (see methods).

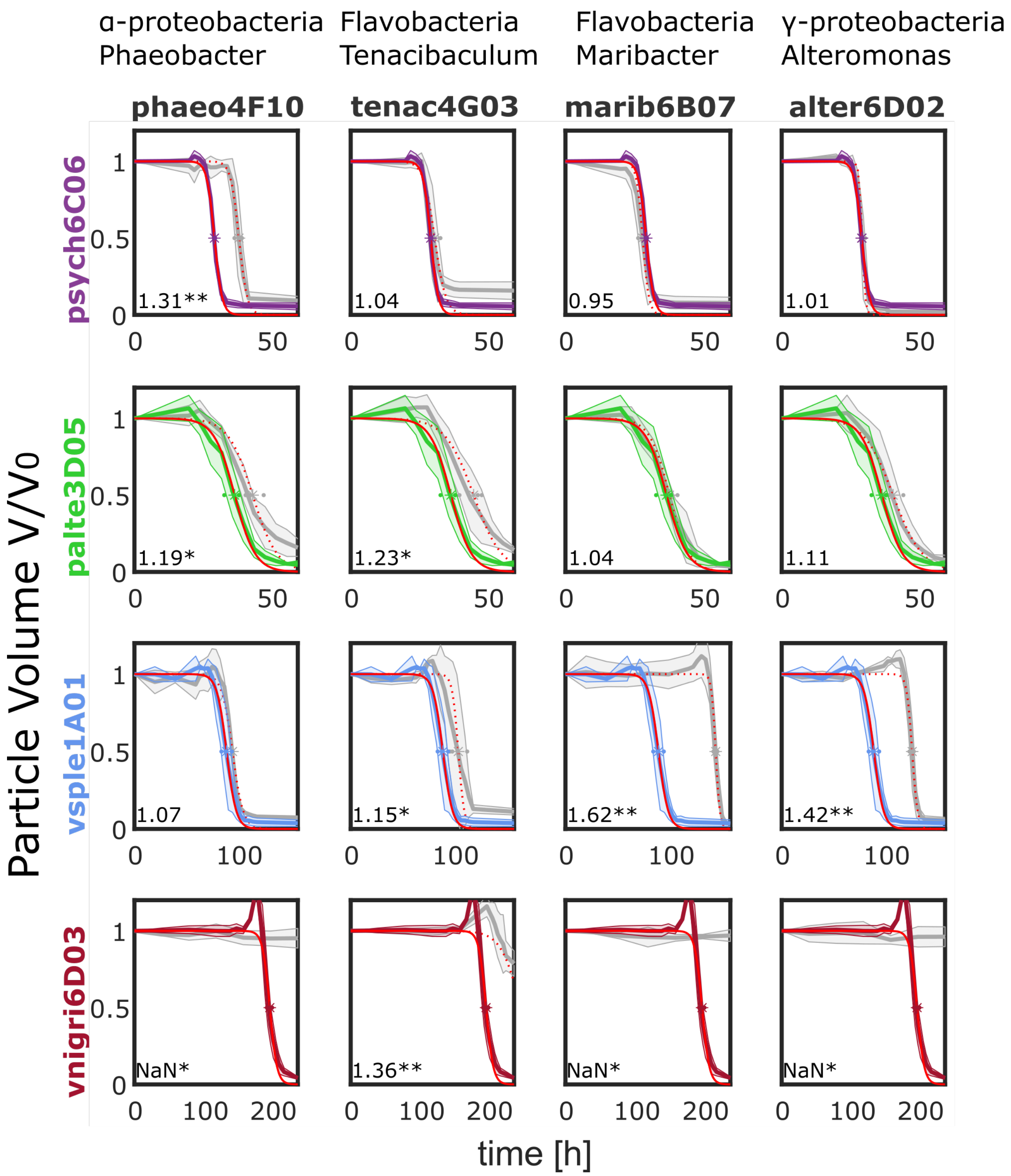

Supplementary Figure 9: Data underlying the network in Fig. 4: Each panel depicts the normalised particle degradation over time for the primary degrader (row) and the secondary degrader (colum) in the respective colors indicated on the strain name. Solid line: mean, shading: standard deviation, n=3. Red solid line: sigmoidal fit to infer $\tau_{1/2}$ for the respective primary degrader, red dashed line sigmoidal fit to infer $\tau_{1/2}$ for the respective co-culture. Asterisk indicates the values of each replicate for $\tau_{1/2}$. Text bottom left indicates the ratio of to $\frac{\tau_{1/2\ co-culture}}{\tau_{1/2\ mono-culture}}$ used to infer the edge thickness of the network in figure 4 and black asterisks indicate significance levels, * < 0.05, ** < 0.1. See also Table S3.

Supplementary Tables

Supplementary Table 1: Genomic features of chitin degraders (in red) and non-degraders. Chitin degraders tend to have multiple copies of chitinases, as well as chitin binding proteins, GlcNAc chemotaxis and PTS transporter genes. The genomes are deposited at NCBI under Bioproject # PRJNA414740 and the respective accession numbers below.

Strain	# chitinases, Chi	# chitin binding proteins, ChB	# GlcNAc specific chemotaxis genes, GTx	# GlcNAc specific PTS transporter	Accession nr.
psych6C06	19	0	1	2	PIZM00000000
vsple1A01	5	2	7	2	PDUR00000000
palte3D05	7	1	2	0	PDUS00000000
vnigr6D03	10	0	5	2	PIZL00000000
marib6B07	2	0	0	0	PDUT00000000
rhodo4F10	6	0	0	0	PDUV00000000
tenac4G03	3	0	0	0	PDUU00000000
alter6D02	1	1	1	0	PIZK00000000

Supplementary Table 2: R^2 and p-value of the multiple linear regression from Figure 2E.

	R^2	p-value
psych6C06	0.96	5.63E-13
palte3D05	0.82	1.97E-07
vpsle1A01	0.78	1.46E-05
vnigri6D03	0.40	4.88E-03

Supplementary Table 3: Data and statistics used to create the network in Fig 4. Tau_ratio and pvalue are used to draw significant edges and determine edge thickness.

primary_degrader	secondary_consumer	mean_tau_primary_degrader [h]	std_tau_primary_degrader [h]	mean_tau_co_culture [h]	std_tau_co_culture [h]	tau_ratio	df_between	df_within	F	pvalue
6C06	4F10	29.41	0.22	38.39	1.44	1.31	1	4	113.46	0.00044
6C06	4G03	29.41	0.22	30.72	1.98	1.04	1	4	1.29	0.31964
6C06	6B07	29.41	0.22	27.98	1.62	0.95	1	4	2.31	0.20326
6C06	6D02	29.41	0.22	29.58	0.92	1.01	1	4	0.09	0.77591
3D05	4F10	36.59	3.12	43.5	3.51	1.19	1	4	6.48	0.06359
3D05	4G03	36.59	3.12	44.92	4.37	1.23	1	4	7.22	0.05483
3D05	6B07	36.59	3.12	37.97	2.43	1.04	1	4	0.37	0.57806
3D05	6D02	36.59	3.12	40.71	3.49	1.11	1	4	2.33	0.2018
1A01	4F10	89.94	5.16	95.94	0.42	1.07	1	4	4.04	0.11495
1A01	4G03	89.94	5.16	103.59	9.18	1.15	1	4	5.05	0.08802
1A01	6B07	89.94	5.16	145.62	1.94	1.62	1	4	305.87	6.00E-05
1A01	6D02	89.94	5.16	127.74	2.26	1.42	1	4	134.9	0.00031
6D03	4F10	198.66	3.85	NaN	NaN	NaN	0	2	NaN	NaN
6D03	4G03	198.66	3.85	270.01	34.51	1.36	1	4	12.67	0.02361
6D03	6B07	198.66	3.85	NaN	NaN	NaN	0	2	NaN	NaN
6D03	6D02	198.66	3.85	NaN	NaN	NaN	0	2	NaN	NaN
