# Supplementary material for: Microscale ecology regulates particulate organic matter turnover in model marine microbial communities
Source: Nat Commun. 2018 Jul 16;9:2743. doi: 10.1038/s41467-018-05159-8 (PMC6048024; doi:10.1038/s41467-018-05159-8)
Supplement: Supplementary file 2 — Description of Additional Supplementary Files [file 41467_2018_5159_MOESM2_ESM.pdf]

Description of Additional Supplementary Files

File Name: Supplementary Movie 1
Description: 3D reconstruction of a chitin micro particle colonized by palte3D05 for 24 h and stained with SYTO9.

File Name: Supplementary Movie 1
Description: Phase contrast time-lapse of a chitin particle cross section taken during degradation by vsple1A01 and corresponding to the micrographs shown in Figure 1C.
